# Supplementary material for: TaeC: A manually annotated text dataset for trait and phenotype extraction and entity linking in wheat breeding literature
Source: PLoS One. 2024 Jun 13;19(6):e0305475. doi: 10.1371/journal.pone.0305475 (PMC11175518; doi:10.1371/journal.pone.0305475)
Supplement: Attachment — Submitted filename: Response to Reviewers.docx [file pone.0305475.s001.docx]

**'Response to Reviewers'**

We would like to thank all the reviewers and the editor for their thorough reviews, valuable comments and constructive suggestions. We are committed to addressing the concerns raised and have made significant revisions to our manuscript to enhance clarity, depth, and the overall contribution of our work. We hope these revisions substantially improve our manuscript and further contribute to the field.

Reviewer #1

*1. I believe the study is not properly placed into the context of previous publications and has failed to acknowledge highly relevant studies. The aim of the authors was to use text analytics to make the large amount of literature on genotype-phenotype relations in wheat more accessible and searchable. I believe this goal is very much at the heart of “knetminer”, which intertwines vast amounts of data from a spectrum of species, to bridge the gap between the considerable amount of available information from publications and scientific discovery.*

We thank Reviewer#1 for their valuable comments. We appreciate their insights and have taken them into consideration for the revision of our manuscript. We have extended the description of the context of our work to include crop information networks such as Knetminer, WheatIS and AgroLD. We have added a paragraph (lines 59 to 85) on this topic in the revised version of the manuscript.

*Knetminer has also used text analytics and has already achieved a very robust information network, which is easily accessible via the web-based interphase. Furthermore a specific wheat knetminer network has been published already. Without the authors comparing their achievements to knetminer, and pointing out the potential advancements of their research in this field, I believe the current manuscript as insufficient for publication.*

The goal of our work in this paper is not at all to present an information network about genotype-phenotype relations in wheat as the specific wheat Knetminer network. Nor is it our aim to present an operational text analysis pipeline to support the creation of such network.

Our achievement is a corpus with phenotypic traits and species manually annotated without the ambition to describe a text analysis pipeline that would use it.

We claim that a corpus like *TaeC* is an essential step of the development of robust text analysis pipelines because it provides the necessary information to train modern machine learning algorithms and to evaluate the quality of existing text analysis pipelines with respect to a gold reference. To the best of our knowledge there is no published training and evaluation corpus that supports any other specific wheat text analysis pipeline.

In particular, the text analysis strategy of KnetMiner is basic, consisting of searching ontology class labels in the documents and it has not evolved since its publication in 2010. It could benefit from using Taec to evaluate the current version and to train up-to-date machine learning methods.

*2. The authors state in the conclusion that “In short-term work, the NER/NEL methods we trained with Taec will be integrated into a AlvisNLP workflow that will be used as a service of the D2KAB project to automatically annotate all PubMed references on wheat.” I believe this short-term goal should be part of the presented study as otherwise an important output/result of this work is missing.*

We agree that this short-term goal is an important output/result of this work. However, we plan to publish the work on the workflow, its training and its evaluation in a separate paper. We believe that our work on *Taec* deserves its own article, as is usually the case with corpus publications. See, for example, the corpora cited in the papers (PGR, Knownleaf, SeeDev, LINNAEUS COPIOUS, S1000). In Plos One, see for example <https://doi.org/10.1371/journal.pone.0283622> <https://doi.org/10.1371/journal.pone.0280637> <https://doi.org/10.1371/journal.pone.0221582>, <https://doi.org/10.5281/zenodo.4526477>, <https://doi.org/10.1371/journal.pone.0079517>

*3. Overall, the manuscript is not up to standards regarding the structure and formatting of text, tables, and figures. I believe the manuscript must be overall improved before publication. Most importantly, the manuscript is missing a discussion. The current discussion (line 524-532) only describes the problem of using text analytics for wheat varieties. It does not discuss the presented results, or places these in the context of current literature.*

We thank Rewiever#1 for their valuable comments. The discussion has been expanded as suggested and it now discusses the presented results, their potential and limitations (lines 587 to 640).

*I have listed all other concerns regarding style and structure under the minor revision section.*

Minor revisions:

*1. As mentioned under the major revision point 3, the sections are not well structured. The introduction is not very concise and could be improved*

We have rewritten the Introduction as recommended and we hope it makes it more concise.

*furthermore the results and methods are not well organized, for example: From line 509, The following section is methods and should thus be moved to the relevant section, rather than appearing in results section.*

We have moved the paragraph about the methods and parameters to the Method section (lines 328 to 343), as suggested by Reviewer#1.

We have also move the method description from the Results section to the Methods section.

*2. TaeC spelling not consistent throughout text (varying between Taec/TaeC, sometimes italicized). Please correct throughout manuscript.*

TaeC spelling has been consistently revised throughout the manuscript.

*3. In multiple incidences words are capitalized in the middle of the sentence (incorrectly), for example line 130. Please change this throughout manuscript.*

We have carefully checked the capitalization of words throughout the manuscript and have revised the capitalization where appropriate.

*4. Table 3 has un-helpful color scheme, no heading for columns apart from column 1, unnecessary use of # before row entries, use of abbreviations with no explanation (av.), and insufficient explanation in the table heading. However, other tables suffer from some of the same issues and would need to be revised, and standardized within the manuscript.*

We have revised the color scheme of the tables, introduced all abbreviations and expanded the table headings to make them self-sufficient.

*5. The example of “earliness per se” used in figures and text throughout the manuscript might be miss-leading. It is a very simple File.txt entry, which has exactly one trait and one phenotype match in file a1. It also has very clean semantic annotations in file a2. Opening more than 10 random files of the supplemental data I could not find any case of such a clear annotation. Most file a1 contained very long, if not all parts of the text in the txt file. In file a2 multiple, species or phenotype annotations are listed for each text. I believe a more representative example could be chosen, or a section must be added to inform the reader that a simplified example was chosen.*

We thank Reviewer #1 for their relevant suggestion. We have added a more complex and representative example of trait mention that involve causality (Fig2). We have kept the simple earliness example from Fig1 to introduce the concept of semantic annotation. Table 1 shows nine examples of text mentions that are complex to annotate with WTO classes. These examples are text snippets and not full texts for readability reasons. Fig3 shows a screenshot of the annotation editor with many different annotations. Since all the data is provided, we do not want to make the article too cumbersome with more examples than these. Readers can also examine the data for themselves.

*6. One of the examples of ontology given in table 1 is not consistent with the use of the term ontology, nor the other examples: “Number of fertile florets at anthesis” is labeled as “percentage of florets without grain”. I do agree that both terms are related to the same plant structure (florets) and also describe similar phenotypes, however the number of fertile florets at anthesis is not the same as percentage of florets without grain. Anthesis happens before grain set, most florets are fertile at this stage, although not all, and most fertile florets will set grain, albeit not all. And no floret contains grains at anthesis! The percentage of florets without grain is measured later in crop development and the percentage is determined by (1) the percentage of fertile florets at anthesis and (2) the number of these florets that set grain. Could the authors explain if in table one the two items are only ontology-matches because they are related to a similar phenotype, or if indeed the pairs should be synonymous to each other, in which case the floret example would have to be removed.*

We thank Reviewer #1 for their careful reading. The pair does not need to be exact synonymous to each other but at least the annotation should be inferred from the class. This was actually an error. We replaced “percentage of florets without grain” with "fertility". The WTO ontology does not contain a more specific class that would be relevant.

Reviewer #2:

*However, some points are already drawn by the authors which are considered as limitations to the comprehensive scope and applicability of the corpus: TaeC currently lacks annotations for varieties and cultivars, a notable limitation given their significance in wheat breeding. The absence of these entities may restrict the corpus's applicability in capturing the full spectrum of wheat-related research, particularly in contexts where specific varieties or cultivars play a crucial role. Additionally, an inherent bottleneck lies in the intricate nature of annotating complex expressions related to cultivars or accessions. The manual annotation process demands a high level of expertise and resources, making it labor-intensive and potentially limiting scalability. Addressing this bottleneck requires exploring automated or semi-automated annotation approaches to enhance efficiency.*

As highlighted by Reviewer #2 the complexity of manual annotation in very technical domains such as the domain of phenotypical traits explains the scarcity of available corpora in plant biology. To that respect, *Taec* is comparable to other corpora in number of entity types and richness of the semantic sources.

We are well aware of the value of annotation of cultivars and varieties we have come up against the diversity of causes of natural and induced genetic variation (crossbreeding, mutations), which are described by a wealth of linguistic expressions that needs to be studied in their own right. To come to this conclusion, as it is suggested by Reviewer #2 we have deeply explored automated or semi-automated annotation approaches to enhance the efficiency of cultivar or accession recognition. For that purpose, we have used the catalogues of the commercialized accessions and the accession names of the *Genetic Resources Information System for Wheat and Triticale database* (GRIS). The results obtained were too noisy and incomplete to be used to speed up manual annotation. We have added this explanation to the Discussion section (lines 615 to 637).

*While TaeC excels in annotating traits, phenotypes, and species, there's a need for explicit discussion on the corpus's scope. Clearly defining the intended applications and potential constraints ensures a better understanding of TaeC's generalizability and use cases. This clarification is vital for researchers and practitioners leveraging the corpus for various applications within the field of cultivated plant phenotyping.*

We regret that the scope of the corpus was not clear to Reviewer #2. We have added some clarifications in the abstract, introduction and discussion about wheat information networks that would benefit from using *Taec*, which we hope will satisfy the request.
